# Study on burgers vector of dislocations in KDP (010) faces and screw dislocation growth mechanism of (101) faces

**DOI:** 10.1039/d0ra08968k

**Published:** 2021-02-18

**Authors:** Bo Yu, Longyun Xu, Shenglai Wang, Pingping Huang, Hui Liu, Liyuan Zhang, Xianglin Li, Bo Wang, Guangwei Yu, Tingting Sui

**Affiliations:** State Key Laboratory of Crystal Materials, Crystal Institute, Shandong University Jinan 250100 China slwang67@sdu.edu.cn; Key Laboratory of Functional Crystal Materials and Device, Ministry of Education, Shandong University Jinan 250100 China; Hunan Province Engineering Technology Research Center of Uranium Tailings Treatment, School of Resource Environment and Safety Engineering, University of South China Hengyang 421001 China

## Abstract

We modified the conventional etching-optical method to measure dislocation direction in a KDP crystal. As burgers vector of dislocation in the KDP crystal must match the minimum periodic vector of the crystal lattice, we suggest that dislocations with a burgers vector of [101], [102] and [103] exist. Atomic force microscopy was employed to characterize the morphology of growth spirals on the hillock of (101) faces. Multi-spirals consisting of more than two element steps with a height of 0.5 nm which is equal to (101) face interplanar distances were observed. We propose the multi-spiral structure is determined by the burgers vector of the corresponding dislocation, and constructed a geometric model of the crystal with screw dislocation to derive the relationship. Growth spirals on the (101) face present a particular triangular morphology and we proved that the triangle structure is formed by connected steps in the 1/2[111] and [010] direction. Micropipes form when the magnitude of the dislocation's burgers vector exceeds 1 nm, as predicted by BCF theory. Interaction between dislocations was observed also.

## Abstract 2

We modified the conventional etching-optical method to measure dislocation direction in a KDP crystal.

## Graphical abstract

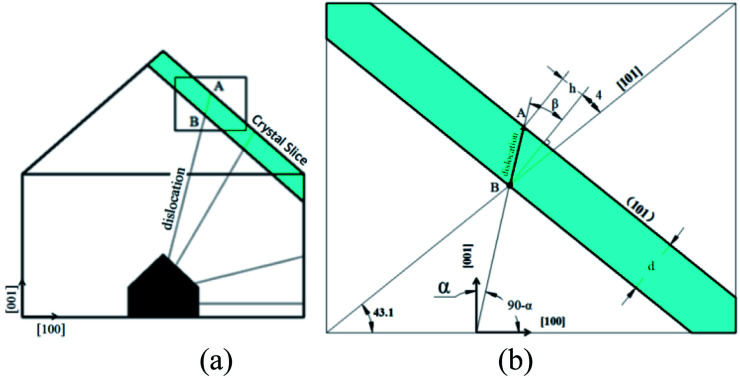

## 1 Introduction

A KDP/DKDP crystal is an excellent non-linear material and also the unique material used to construct the frequency converter on an inertial confinement fusion system (ICF).^1,2^ The ICF laser with high intensity requires single crystal boules with large scale and high optical quality. To meet the requirement, investigation of the crystal growth mechanism at the micro level is necessary.

Dislocation is a common linear defect in crystals. Existence of dislocations would damage the homogeneity of the material and causes interior stress.^3,4^ However, crystal growth can also benefit from dislocations by screw dislocation growth mechanism, *i.e.* the Burton–Cabrera–Frank (BCF) theory.^5,6^ Direction of dislocation in KDP has been intensively investigated by optical method and synchrotron radiation technique, and researchers categorize dislocations in the (010) face into three types as with a burgers vector of [001], [101], and [100].^7,8^ But this is far from being a convincing conclusion. The discrete property of dislocation determines that distribution of dislocation direction should concentrate in its burgers vector's direction. But all reported dislocation distributes in a wide range. It's unreasonable to assert all these dislocations have the same burger vector.

According to screw dislocation growth mechanism, crystal growth is a process that growth units attach to the steps on crystal surface caused by screw dislocation. The movement of the step around the dislocation would finally leave a growth spiral on the surface.^6^ De Yoreo has observed multi-spiral consisting of several element steps with triangular morphology on (101) faces.^9^ But further work is needed to explain how the triangular morphology and the multi-spiral structure forms.

In our work, modified optical method was used to measure the direction of KDP dislocation. Considering the discrete property of dislocations, we suggest that there exist dislocations with burgers vector of [102], [103]. Hillocks on (101) faces were characterized by atomic force microscope (AFM), morphology of the triangular multi-spirals observed are described fully. The mechanism of formation of the triangular morphology and the multi-spiral is explained. Dimensions of micropipes found at the dislocation source are measured. At last, interaction between dislocations was discussed.

## 2 Experiments

### 2.1 Crystal growth and characterization of the growth spirals on KDP (101) face

Crystals are grown rapidly by point seed technique, carbon tetrachloride is used to preserve the surface. Growth spirals were characterized by scanning hillock on (101) faces with AFM equipment (Dimension Icon with ScanAsyst, Bruker). Height images obtained are 1st flattened.

### 2.2 Optical method to measure the dislocation direction in KDP crystal

**Fig. 1:**
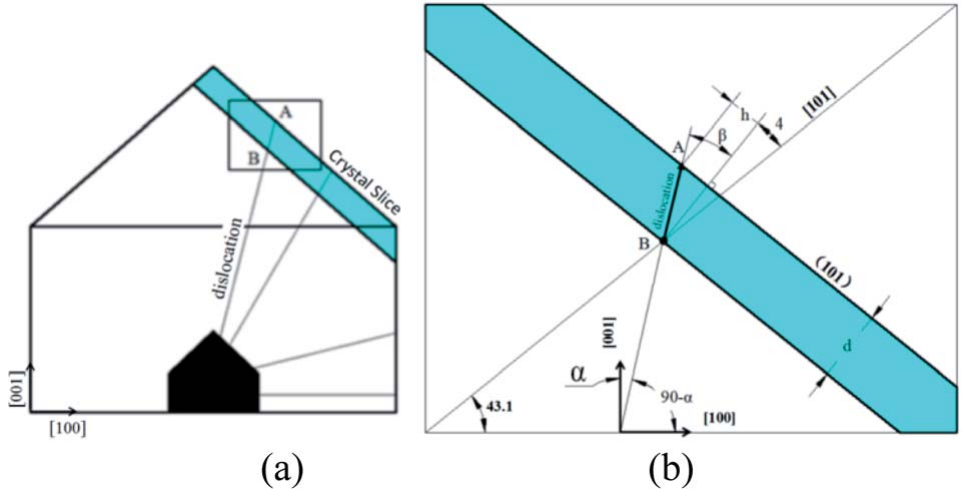
(a) Dislocations in (010) face of KDP crystal by point seed technique; (b) illustration for measurement of dislocation direction.

The cut crystal slices in (101) direction as shown in Fig. 1(a). Polish both surfaces until no mechanic damage visible to human eyes. Then etch slices by immersing into deionized water for 3–5 s, take out and wipe them dry with cotton softly.

X-ray topography and synchrotron radiation proves that the geometric characteristic of dislocation in KDP crystal is a straight line originates from the seed source until reach crystal surface, as shown in Fig. 1(a).^3,7^ Triangular pyramid pit would form on the surface where the dislocation terminates by chemical etching.^10^ Thus, method to measure the dislocation direction is designed, as shown in Fig. 1(b).

In Fig. 1(b), width and height of rectangle presents [100] and [001] direction, the block in green is the crystal slice in (101) direction with a thickness of *d*. Dislocation line BA has an angle of *α* respect to *c* axis lays in (010) face and penetrates crystal slice. After etching, pits left on both A and B point on the upper and lower surface where dislocation terminates. The projection distance *h* of A and B can be measured by optical microscope, so the angle *β* between the dislocation line and the normal of (101) face can be calculated as *β* = arctan(*h*/*d*). Finally, by geometrical relationship we have 90.0° − *α* = 43.1° + *β* + 3.8°, *i.e.*:

1 *α* = 43.1° − arctan(*h*/*d*)

## 3 Results and discussion

In this paper, we use below convention to define a screw dislocation right-handed or left-handed. Put your right hand thumb align with the normal direction out of the crystal surface, when the curl of other fingers aligns with the outward direction of growth spiral, the dislocation is right-handed, or it is left-handed.

### 3.1 Morphology of growth spiral on KDP (101) faces

**Fig. 2:**
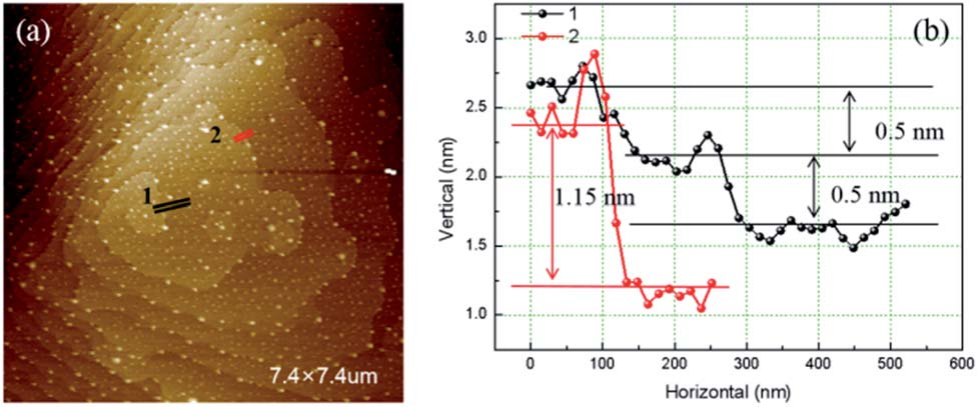
(a) AFM image of hillocks with *b*_⊥_ = 2*h* (b) cross sections profile along segments.

In Fig. 2 is a right-handed growth spiral with 2 element steps (hillocks with *b*_⊥_ = 2*h*). Follow the dark segment a slight separation of bunched steps happened. The profiles of cross section along segments are presented in right. The step heights measured along red segment (bunched step) is 1.15 nm, while along the black bunched step separate into two element steps of 0.5 nm height.^11^ Element steps of the hillocks with *b*_⊥_ = 2*h* are bunched together in most area, makes it look as a single spiral.

**Fig. 3:**
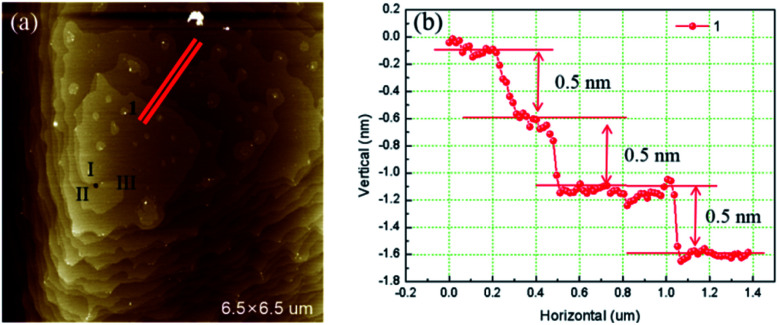
(a) AFM image of hillocks with *b*_⊥_ = 3*h* (b) cross section along red segment.

Fig. 3 is the AFM image of a left-handed hillocks with *b*_⊥_ = 3*h* consists of three totally separated element steps. The profile of cross section along the blue segment shows that all the height of the element steps are 0.5 nm. At the dislocation source, a cylindrical micropipe predicted by BCF theory is found.^9^ The average diameter of the micropipe measured from two perpendicular directions is 73 nm.

**Fig. 4:**
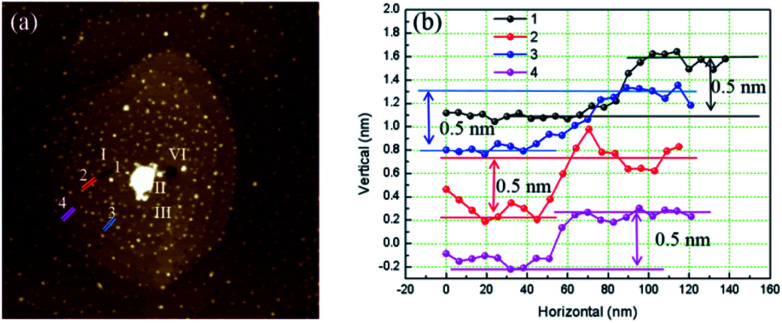
(a) AFM image of hillocks with *b*_⊥_ = 4*h* (b) cross section along different colors segments.

Fig. 4 is the AFM image of a left-handed hillocks with *b*_⊥_ = 4*h*. The sample surface was severely contaminated by the deposited crystals but still four totally separated element steps can be identified. All the heights of element step measured are 0.5 nm. Micropipe is found at the dislocation source as well, and the average diameter is 140 nm.

**Fig. 5:**
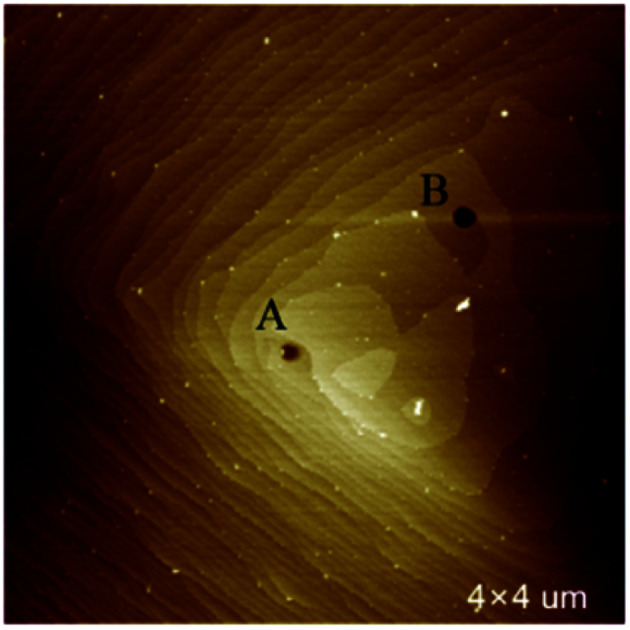
Two interacting dislocations.

Except isolated dislocations with 2, 3, 4 element step, complex dislocation sources with more than two close dislocations were also observed. In Fig. 5 presents two interacting dislocations, the dislocations are right-handed, the interaction between them is repulsion. Micropipes are existed at the dislocation sources. The average diameter measured for each micropipe is: A 90 nm, B 145 nm.

### 3.2 Dislocation directions measured by optical method

**Fig. 6:**
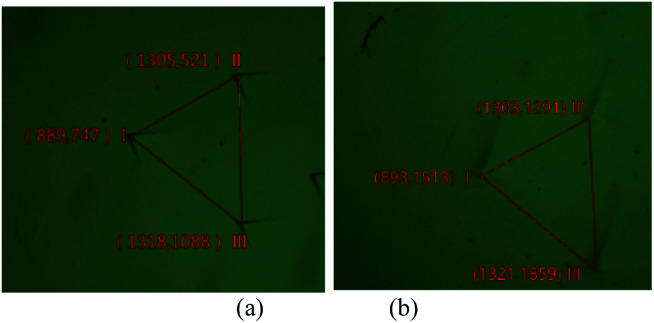
Pits caused by the same bunch of dislocations on upper (a) and lower (b) surface and their image coordinates, the scale of image is: 278.55 μm/100 pixel.

In Fig. 6 are the images of upper surface and lower surface of an etched crystal slice. Two groups of etching pits have the same triangular pattern are highlighted by red triangles. These two groups of pits were identified as formed by the same bunch of dislocations. We have chosen the vertexes to represent the location of etching pits and labelled their image coordinates correspondingly.

The relative distance (in mm) calculated for corresponding pits are as Table 1:

**Table 1:** Relative distance of dislocation pits

Etching pit	I–I′	II–II′	III–III′
Horizontal distance	0.01	−0.01	−0.01
Longitudinal distance	2.14	2.13	2.13

The data calculated for the three dislocations are almost the same, thus all the dislocations have the same direction.

Horizontal distance can be regarded as 0, which means the dislocations lay in (100) face.

By substituting *h* into formulate (1) the angle between dislocation line and *c* axis *α*_I–I′_ = 19.9°, *α*_II–II′_ = 19.9°, *α*_III–III′_ = 20.0°.

Totally, 6 groups of measured data were obtained, as shown in column 2, Table 2. All the dislocation observed lay in [010] face. Here direction of dislocation is the angle between dislocation line and *c* axis in degrees.

**Table 2:** The direction of dislocation measured by optical method

No.	Measured direction	Corresponding theoretic dislocation				Variation	Reported data from former research		
		Burgers vector	Direction	Magnitude	Number of element steps		Data from J. C. Chen^9^	Data from Klapper^10^	Burgers vector
1	19.9	[013]	19.6	2.22	4	0.3	12–15	12–21	[001]
2	19.9					0.3			
3	20.0					0.4			
4	28.3	[012]	28.1	1.58	3	0.2	Inconsistent with references		
5	27.9					−0.2			
6	28.4					0.3			
7	46.7	[011]	46.9	1.02	2	−0.2	46–50	43–47	[011]
8	47.4					0.5			
Not observed							78	75–76	[010]

In our experiment we only observed dislocations lay in (010) face. In subsequent discussion, we confine the dislocations discussed lay in (010) face.

## 4 Discussion

### 4.1 Burgers vector of dislocation and formation of multi-spiral structure

Burgers vector for perfect dislocation in crystal can only be one of the minimum periodic vectors of crystal lattice to meet the lowest stain energy requirement.^3^

**Fig. 7:**
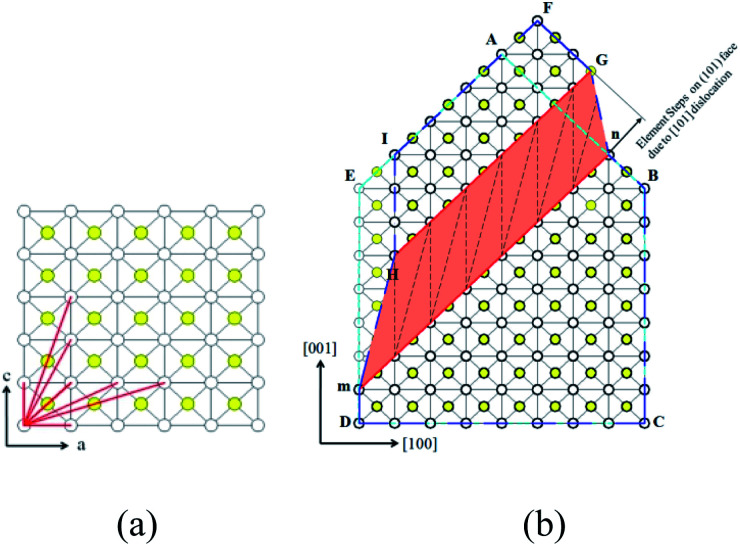
(a) Lattice structure of (010) face of KDP crystal (b) steps on (101) face caused by [101] dislocation. In figure (a), in clockwise direction red segment represent dislocation of [001], [103], [102], [101], [201], [301], [100].

Fig. 7(a) is the lattice structure of (010) face, lattice sites are presented by dots. KDP crystal is body-centered structure, white dots represent the lattice sites locate at corner, while the yellow dots represent the lattice sites locate at body center. The red segments represent some of the minimum periodic vectors, from which the burgers vectors of dislocations can be. The direction and magnitude of these burgers vectors are listed in Table 2.

In Fig. 7(b), we introduce a screw dislocation with burgers vector of *b* = [101] to investigate how dislocation alternate crystal lattice. ABCDE represents the perfect crystal without dislocation. mn is the dislocation line of the screw dislocation and the paper surface is glide plane. When the area surrounded by AnmE above the glide plane glides a displacement of *b* = [101], a screw dislocation with burgers vector [101] is formed. The deformed shape of crystal above glide plane is FGnBCmHI. Area of HmnG is called distortion area where almost of the strain energy stored, also where micropipe forms.

Obviously, screw dislocation of [101] caused a bunched step onto (101) face, the step can be split into two element steps with height equal to the distance of single atom layer in (101) direction, this explains why the element step height measured on (101) face is 0.5 nm.^11^ We also found the number of element steps is equal to the number of single atom layers the screw dislocation penetrates. For the hillocks with *b*_⊥_ = 2*h* case in Fig. 7(b), it can be formed by dislocation of [101], because [101] dislocation penetrates 2 atom layers and forms two element steps on (101) face.

When element steps are subjected to supersaturation, each would be activated and move forward around the dislocation line, then multi-spiral forms.^12^ In column 6, Table 2 lists the number of element steps for some screw dislocations. The hillocks with *b*_⊥_ = 2*h* can only form by dislocations of [101], hillocks with *b*_⊥_ = 3*h* and hillocks with *b*_⊥_ = 4*h* can form by [102] and [103] or [201] and [301].

Now we go back to the dislocation direction obtained by optical method. The measured direction of no. 1–3 are close to the direction of [103] dislocation, no. 4–6 are close to [102] dislocation, no. 5 and no. 6 are close to [101] dislocation. Except no. 4–6, dislocations assumed to be [102] dislocation, all the data are consistent with references. Considering the multi-spiral and its relationship with dislocation's burgers vector, it is further confirmed that in KDP crystal exist screw dislocations with burgers vector of [102] and [103]. These dislocations result in the growth spiral with multi-spiral structure, and have great impact on the process of crystal growth.

### 4.2 Micropipe

When element step number exceeds 2, micropipe was found around the spiral center at the dislocation source. The profile of axial section of micropipe is approximately a circle. The conical profile of radial section is result of interaction between AFM probe and the wall of micropipe. We measured the diameter of the cone 2–3 nm lower the surface to avoid interruption due to AFM probe and micropipe wall interaction. The average diameter of the micropipe for hillocks with *b*_⊥_ = 3*h* and hillocks with *b*_⊥_ = 4*h* is 73 nm and 140 nm. De Yoreo also observed the same phenomenon and the corresponding value is 62 nm and 136 nm, which is very close to our results.^9^

According to BCF theory, when the norm of a dislocation's burgers vector exceeds a critic value, which is approaching to 1 nm for most material, crystal tends to remove the severely deformed material at the dislocation source to form a hollow core structure.^13^ When a crystal is growing the growth units cannot fill into the core of dislocation during the growth process, a tube will form and extend until reach the crystal surface.

In anisotropic materials, the relationship between radius of micropipe rand burgers vector of dislocation *b* is:^14,15^2
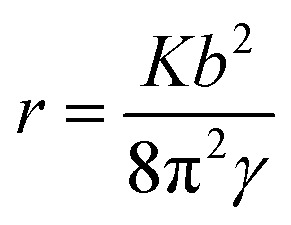
where *K* is the energy factor of the dislocation, *γ* is the average surface energy of the micropipe, both of the two parameters are direction depended.^3^ We have proved burgers vectors of dislocation for hillocks with *b*_⊥_ = 3*h* and hillocks with *b*_⊥_ = 4*h* are [102] and [103], which tilt less than 9° to each other. It's reasonable to assume the parameters *K* and *γ* are equal as an approximate treatment for the two dislocations. Substitute the burger vector into the formula, the micropipe of hillocks with *b*_⊥_ = 4*h* should be 1.97 times the hillocks with *b*_⊥_ = 3*h* as measured, quite close to the value measured.

In Fig. 6, interaction between dislocations is observed. For dislocation B, when we follow one spiral in a full loop, we found there are two layers between loops. This means the burger vector of dislocation B won't exceed [102]. But the diameter of micropipe at dislocation B is 145 nm, much bigger than the diameter of micropipe formed individually by a [102] dislocation. Obliviously, repulsion interaction between dislocations increase the strain energy, make the diameter of micropipe bigger than when it exists individually.

### 4.3 Topography of growth spiral

**Fig. 8:**
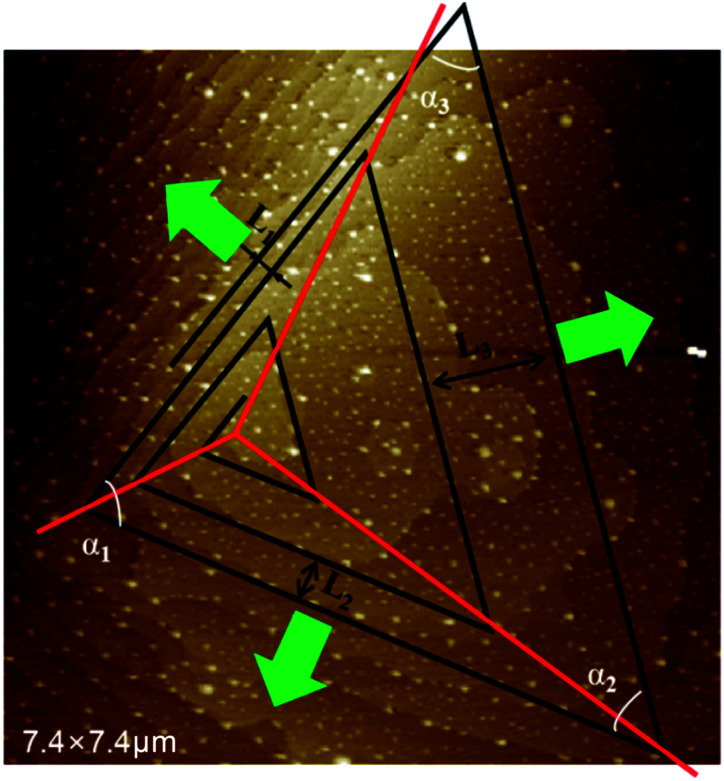
Hillocks with *b*_⊥_ = 2*h* fitted with triangular spiral lines.

Equidistance triangular spiral topography is observed on (101) face of KDP crystal, which is consistent with the theoretic analysis of De Yoreo.^9^ In Fig. 8, straight segments are used to fit the growth spiral, angles between adjacent connected steps and distances between parallel steps are measured as:*α*_1_ = 74°, *α*_2_ = 54°, *α*_3_ = 52°*L*_1_ = 260 nm, *L*_2_ = 520 nm, *L*_3_ = 1350 nm

**Fig. 9:**
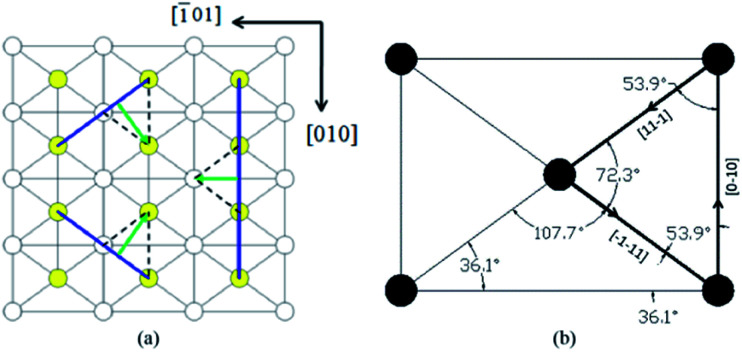
(a) Lattice structure of (101) face (b) values calculated for some angles.

In Fig. 9(a) presented the lattice structure of (101) face. Due to the anisotropy of crystal, the step forms on (101) face it has preferred direction. Bond length for some lattice points are calculated as [101] = 1.020 nm, [010] = 0.698 nm, and 1/2[111] = 0.510 nm. Usually, a shorter the bond length means a strong chemical bond. These periodic chemical bonds in crystal form the periodic bond chains (PBC).^5^ PBC theory defines steps on crystal surface as faces only contain one of the PBCs. Formation of steps along direction of [010], 1/2[111] would release more energy. The green arrows perpendicular to the steps represent moving directions for each step. Black dot lines represent the bonding directions for each step, along which the growth unit is attracted and observed and obviously, 1/2[111] step has the strongest binding force, the following is [010] step, the last is [101] step.

In Fig. 9(b) some angles for (101) face geometry are calculated. In obvious to see that *α*_1_, *α*_2_, *α*_3_ measured for growth spiral is similar to those of the directed triangle enclosed by heavy lines. It is convincible that the spiral on (101) face is evolved from the directed triangle, and constructed by steps along [010], 1/2[111] directions.

Different distances between parallel steps of growth spiral reflect the different moving speeds of steps. Distance between the parallel steps as measured are *L*_1_ = 0.26 nm, *L*_2_ = 0.52 nm, *L*_3_ = 1.35 nm. As in Fig. 8(b), the lattice distance in the step moving direction for step of 1/2[1], 1/2[11] and [00] are 6.32 nm, 6.32 nm and 5.1 nm. Divide the step distance by the corresponding layer distance, obtains layers number each step passed in a certain period, *n*_1_ = 42, *n*_2_ = 82, *n*_3_ = 266, thus ratio of step moving speeds is 1 : 2 : 6.

The formula for normal moving speed of step is:^9^

**Fig. 10:**
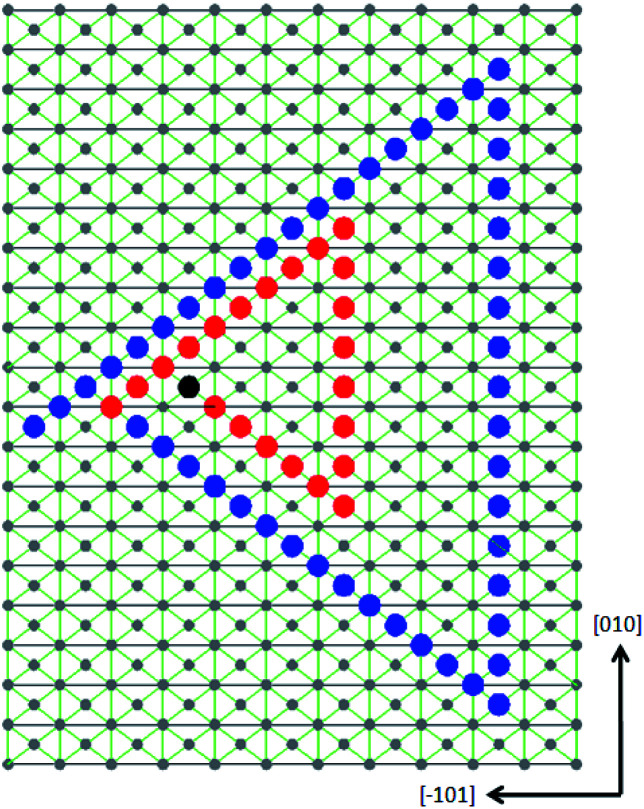
The projection view of the growth spiral on (101) face along the [1̄1̄0] direction. Two growth loops are presented with a moving speed of 1, 2, 6 layers for each step each time. The black dots represent the terminal of screw dislocation. Red dots and blue dots represent the edge of two terraces (steps) has grown on the surface.

3 *v* = *ωβ*(*c* − *c*_0_)

where *ω*, *β* are determined by the intrinsic property and temperature, is determined by the supersaturation. Under the same supersaturation, the ratio of step moving speed is only determined by the intrinsic property of crystal. [010] step has the strongest attraction to the growth units in its moving direction among the three steps, its moving speed is also the highest. Intuitively and steps are symmetrical to (010) plane and should have the same moving speed.^1,9^ But be aware that the unit falls on the lattice point doesn't have the symmetrical property, this results in a different moving speed. Fig. 10 illustrates how the spiral lays on the (101) surface with an simplicity that each of the step passed 1, 2 and 6 layers in a certain period. For multi-spirals, each of the single spirals has exactly the same geometrical structure but with a phase difference.

## 5 Conclusions

In conclusion, our research shows that the growth spiral on KDP (101) surface can be multi-spirals with 2, 3 and 4 element steps. When the element step number excesses 2, a hollow core will form at the dislocation source, and the diameter of the hollow core is positive related with the layer number. Growth spirals on (101) face present a triangular spiral morphology, and the spiral is formed by steps along 1/2[111̄], 1/2[11̄1̄] and [010] directions. Each step has a different moving speed due to the different binding energy, their ratio is about 1 : 2 : 6. Relationship between burgers vector of screw dislocation and element step number of growth spiral is derived for KDP crystal. Based on this relationship, the existence of screw dislocation with burgers vector of [101], [102] and [103] is confirmed.

## Conflicts of interest

There are no conflicts to declare.

## Supplementary Material
